# The impact of chlorhexidine bathing on hospital-acquired bloodstream infections: a systematic review and meta-analysis

**DOI:** 10.1186/s12879-019-4002-7

**Published:** 2019-05-14

**Authors:** Jackson S. Musuuza, Pramod K. Guru, John C. O’Horo, Connie M. Bongiorno, Marc A. Korobkin, Ronald E. Gangnon, Nasia Safdar

**Affiliations:** 1 Department of Medicine, University of Wisconsin School of Medicine and Public Health, Madison, WI USA; 2 William S. Middleton Memorial Veterans Hospital, Madison, WI USA; 3 Department of Critical Care Medicine, Mayo Clinic, Jacksonville, FL USA; 4 Division of Infectious Diseases and Division of Pulmonary and Critical Care Medicine, Department of Medicine, Mayo Clinic, Rochester, MN USA; 5 Bio-Medical Library, University of Minnesota Libraries, Minneapolis, MN USA; 6 University of Wisconsin School of Medicine and Public Health, Madison, WI USA; 7 Department of Biostatistics and Medical Informatics, University of Wisconsin-Madison, Madison, WI USA; 8 Department of Population Health Sciences, University of Wisconsin-Madison, Madison, WI USA

**Keywords:** Chlorhexidine bathing, Hospital-acquired bloodstream infections, Implementation, Intervention fidelity, Patient-centered outcomes, Infection prevention

## Abstract

**Background:**

Chlorhexidine gluconate (CHG) bathing of hospitalized patients may have benefit in reducing hospital-acquired bloodstream infections (HABSIs). However, the magnitude of effect, implementation fidelity, and patient-centered outcomes are unclear. In this meta-analysis, we examined the effect of CHG bathing on prevention of HABSIs and assessed fidelity to implementation of this behavioral intervention.

**Methods:**

We undertook a meta-analysis by searching Medline, EMBASE, CINAHL, Scopus, and Cochrane’s CENTRAL registry from database inception through January 4, 2019 without language restrictions. We included randomized controlled trials, cluster randomized trials and quasi-experimental studies that evaluated the effect of CHG bathing versus a non-CHG comparator for prevention of HABSIs in any adult healthcare setting. Studies of pediatric patients, of pre-surgical CHG use, or without a non-CHG comparison arm were excluded. Outcomes of this study were HABSIs, patient-centered outcomes, such as patient comfort during the bath, and implementation fidelity assessed through five elements: adherence, exposure or dose, quality of the delivery, participant responsiveness, and program differentiation. Three authors independently extracted data and assessed study quality; a random-effects model was used.

**Results:**

We included 26 studies with 861,546 patient-days and 5259 HABSIs. CHG bathing markedly reduced the risk of HABSIs (IRR = 0.59, 95% confidence interval [CI]: 0.52–0.68). The effect of CHG bathing was consistent within subgroups: randomized (0.67, 95% CI: 0.53–0.85) vs. non-randomized studies (0.54, 95% CI: 0.44–0.65), bundled (0.66, 95% CI: 0.62–0.70) vs. non-bundled interventions (0.51, 95% CI: 0.39–0.68), CHG impregnated wipes (0.63, 95% CI: 0.55–0.73) vs. CHG solution (0.41, 95% CI: 0.26–0.64), and intensive care unit (ICU) (0.58, 95% CI: 0.49–0.68) vs. non-ICU settings (0.56, 95% CI: 0.38–0.83). Only three studies reported all five measures of fidelity, and ten studies did not report any patient-centered outcomes.

**Conclusions:**

Patient bathing with CHG significantly reduced the incidence of HABSIs in both ICU and non-ICU settings. Many studies did not report fidelity to the intervention or patient-centered outcomes. For sustainability and replicability essential for effective implementation, fidelity assessment that goes beyond whether a patient received an intervention or not should be standard practice particularly for complex behavioral interventions such as CHG bathing.

**Trial registration:**

Study registration with PROSPERO CRD42015032523.

**Electronic supplementary material:**

The online version of this article (10.1186/s12879-019-4002-7) contains supplementary material, which is available to authorized users.

## Background

Hospital-acquired bloodstream infections (HABSIs) are associated with increased morbidity, mortality, length of hospital stay, and costs [1, 2]. Central line-associated bloodstream infections (CLABSIs) account for the vast majority of HABSIs [3]. In the past decade, a number of interventions have led to an overall decline in CLABSI rates in intensive care units (ICUs). These include catheter insertion bundles or checklists [4], disinfection of hubs and needleless connectors [5], and use of chlorhexidine gluconate (CHG) impregnated dressings [6]. More recently, bathing of patients with CHG has received attention as a novel strategy to prevent HABSIs, both CLABSIs and non-CLABSIs [7]. Bathing with CHG may reduce the risk of HABSI by lowering microbial burden on the patient’s skin and the hands of healthcare workers [8–10].

Although some studies have shown the efficacy of CHG bathing in reducing HABSIs, particularly in the ICU, there is considerable variation in the implementation of this behavioral intervention, especially ensuring intervention fidelity. Failure to ensure fidelity to this intervention due to the possible suboptimal compliance with daily CHG bathing can potentially lead to decreased susceptibility of bacteria to CHG and eventual development of resistance [11].

In addition, CHG studies have not addressed patient-centered outcomes [12]; these outcomes are particularly relevant for CHG bathing where the patient may be an active participant. Examples include patient comfort during bathing and CHG-related adverse events, such as skin irritation and dryness, which may affect patient acceptance of the intervention [13–15].

Current infection prevention literature lacks meta-analyses/systematic reviews assessing intervention fidelity and patient-centered outcomes among patients receiving CHG bathing. Given several studies on CHG bathing and its potential for reducing HABSIs, we undertook a systematic review and meta-analysis to examine 1) the magnitude of effect of CHG bathing in different settings, 2) fidelity to the intervention, and 3) patient-centered outcomes.

## Methods

We conducted this systematic review and meta-analysis in conformity with PRISMA and MOOSE guidelines and registered the protocol with the PROSPERO: CRD42015032523 [16, 17].

### Data sources and search strategy

With the assistance of a reference librarian (CMB), we conducted a search for CHG and hospital-acquired infection human studies published through January 4, 2019 without date or language restrictions. We searched Medline (via Ovid), EMBASE, CINAHL, Scopus, and Cochrane’s CENTRAL registry. We used controlled vocabulary or MeSH (Medical Subject Heading) terms in addition to keywords, including “Baths,” “Chlorhexidine,” “Disinfection,” “Soaps,” “Anti-Infective Agents,” “Treatment Outcome,” “Disinfectants,” “Cross Infection,” “Drug Resistance,” “Catheter-Related Infections,” and “Bacteria.” Additional records were identified by reviewing reference lists of included articles.

### Study selection

#### Inclusion and exclusion criteria

Three authors (JCO, JSM, and PKG) assessed eligibility for inclusion of studies in this meta-analysis. Disagreements regarding study inclusion were resolved through discussion. We included randomized controlled trials (RCT), cluster randomized trials (CRT), and quasi-experimental studies that evaluated the effect of CHG bathing versus a non-CHG comparator for prevention of HABSIs in any adult healthcare setting. Studies that compared post-intervention rates with historical controls and review papers were excluded. We also excluded studies of pediatric patients, those that studied pre-surgical CHG use, and those without a non-CHG comparison arm.

#### Data extraction and quality assessment

Two authors (JCO and JSM) independently extracted the data. The following variables were abstracted: first author and year of publication, study design, country, setting, intervention, comparator, study duration, method used to assess fidelity, fidelity components (adherence, exposure or dose, quality of the delivery, participant responsiveness, and program differentiation), patient-centered outcomes assessed in the study, demographics, patient-days at risk, number of HABSIs in the intervention and comparator groups, and intervention bundling (i.e., CHG bathing combined with other interventions). In these bundled interventions, CHG bathing was the prominent component of the bundle, and we performed subgroup analyses comparing bundled vs. non-bundled interventions.

We assessed the quality of studies using a modified version of the Cochrane’s Risk of Bias tool [18]. The domains assessed were subject allocation (e.g., randomized vs. non-randomized), completeness of outcome data, method of outcome assessment (blinded or not), diagnostic criteria for bloodstream infection, and other sources of bias, such as exclusion of certain study subjects during the analysis and information bias during data collection. We qualitatively scored studies as “high” or “low” risk of bias in each of these domains. Three authors (JCO, JSM, and PKG) independently reviewed and assessed each study, and differences in assessments were reconciled via discussion. Reporting and publication bias was assessed using a funnel plot and Egger’s test.

#### Outcomes

The primary clinical outcome of interest was the incidence of HABSIs. The incidence rate ratio (IRR) of HABSIs was calculated as the ratio between the incidence rate (i.e., the number of bloodstream infections identified per 1000 patient-days) among patients treated with CHG vs. that of patients in the control group, or the ratio of the incidence rate of bloodstream infections before and after implementation of CHG bathing. We collected data on causative microorganisms of reported HABSIs and categorized the organisms as fungi (mainly yeasts), gram-negative bacteria, coagulase-negative staphylococci (CoNS), and gram-positive bacteria other than CoNS. We treated CoNS as a separate category as it is a common contaminant [19].

We also assessed intervention fidelity. Fidelity is defined as “the demonstration that an experimental manipulation is conducted as planned” [20]. Dane and Schneider’s proposed five components of fidelity, including adherence, exposure or dose, quality of the delivery, participant responsiveness, and program differentiation, were assessed [21].

##### Adherence

Adherence measures the extent to which the implemented program elements align with the intervention as outlined in the protocol and can be assessed by identifying the primary components of a given intervention. For CHG bathing, this is whether bathing actually occurs and could have been assessed through direct observations of bathing, assessment of CHG purchase, or usage data.

##### Exposure or dose

This measures how much of the program content actually reaches the intended participants (i.e., healthcare workers conducting the CHG baths). For example, program content can include the number of CHG training sessions including their duration and frequency that are completed by healthcare workers prior to implementing CHG use. Exposure does not refer to how much CHG a patient was exposed to during the bath, which is assessed by both adherence and quality of delivery.

##### Quality of the delivery

This assesses the processes and content of an intervention. For CHG bathing, quality of the delivery can be assessed by conducting direct observations of the process to assess if all the bathing steps are followed.

##### Participant responsiveness

Measuring how engaged participants are in a CHG intervention and their perceptions of the intervention involves obtaining feedback from providers administering baths and patients receiving the baths through surveys or interviews.

##### Program differentiation

This assesses the specific ways by which researchers carried out interventions and any unique characteristics. For example, studies should unambiguously report how unit leadership was engaged, if audits were conducted, or how feedback was provided to staff conducting CHG baths. In addition, studies need to clearly state the CHG product used and in what concentration.

In addition, we examined whether studies assessed or reported patient-centered outcomes. These may include major barriers to bathing, such as patient comfort during the bath, adverse events related to CHG and CHG’s lack of a fragrant scent which has especially been associated with patient refusal of CHG baths [13–15]. These outcomes can be passively reported by patients or actively elicited. The patient-centered outcomes we assessed in this study included 1) patient discomfort; 2) smell of the CHG soap and whether this was acceptable to patients; 3) patient education about CHG bathing and 4) adverse events related to CHG bathing, such as skin rashes, skin dryness and pruritus. These were generated through several collaborative discussions with a panel of seven patients at our institution, all of whom have experience with healthcare-associated infections (HAIs) and CHG bathing.

### Statistical methods

The effect of CHG bathing was calculated as the IRR for each study using a continuity correction. The DerSimonian and Laird method was used to obtain estimates of the average intervention effect and the heterogeneity of intervention effects across studies using a random-effects model [22]. We evaluated heterogeneity of the IRR across studies using the I^2^ statistic [23]. As a robustness check, we also estimated the parameters of the random-effects logistic regression model via maximum likelihood. We calculated infection risk using patient-days at risk or venous catheter-days as the denominator depending on whichever the study provided with a preference for catheter-days if both were provided and the outcome was limited to patients with venous access devices. We decided to combine studies that used patient-days at risk and those that used venous catheter-days at risk as the denominator because conducting the analyses separately did not make a difference in terms of the CHG effect. Moreover, the use of patient days in some studies would bias the results towards the null. One study did not report person-time data (patient-days at risk or venous catheter-days) and was omitted from the analysis using person-time as the denominator [24]. To assess the effect of excluding this study, we also conducted a separate analysis using number of patients as the denominator for those studies that reported this information. We conducted subgroup analyses that were defined a priori for the following groups: RCT or CRT vs. non-randomized studies, bundled vs. non-bundled interventions, CHG wipes vs. CHG solution, and ICU vs. non-ICU setting.

We performed statistical analyses using the command “METAN,” with the cc option in Stata software, version 14.0 (Stata Corp. College Station, Texas) and PROC GLIMMIX in SAS 9.4 (SAS Institute Inc., Cary, North Carolina).

## Results

**Fig. 1:**
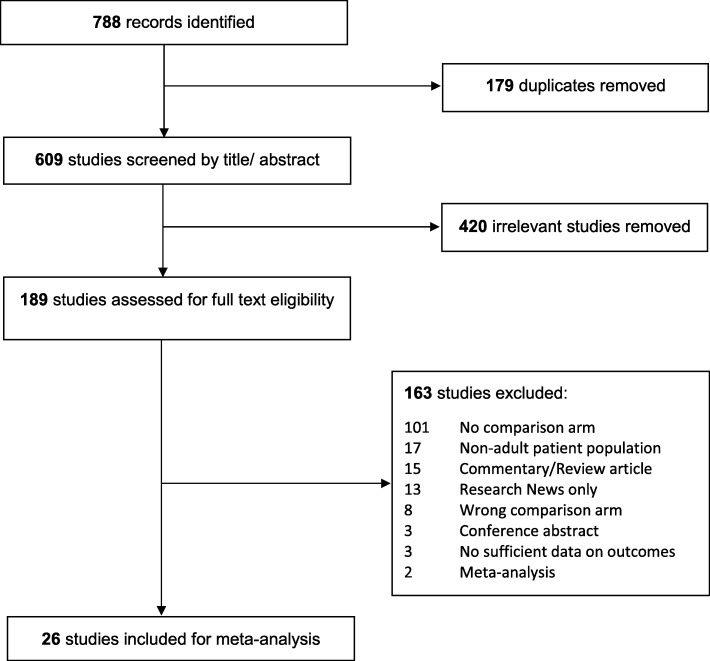
Study selection flow diagram adapted from the Preferred Reporting Items for Systematic Reviews and Meta-analyses (PRISMA)

The search yielded 788 articles of which 179 were duplicates and were excluded. We screened 609 articles. We excluded 420 studies after title and abstract review leaving 189 articles for full article review, after which 163 were excluded. This left 26 articles for the meta-analysis (Fig. 1).

Eighteen studies were non-randomized, and we classified them as quasi-experimental studies; eight were RCTs or CRTs. Most were single-center studies (*n* = 19). Nineteen were conducted in the ICU; the remaining seven were conducted in various settings, such as general medical wards, burns unit, geriatric chronic care units, and long-term acute care hospitals.

The most commonly used product for bathing was the non-rinse 2% CHG wipe (18/27) [8, 25–41]. All but two studies obtained wipes from Sage Product LLC, Cary, IL. For the two studies that did not use Sage cloths, one study used a similar cloth product manufactured by G70 Antisepsis, Mexico City, Mexico [37], and the other used 2% CHG cloths produced by their local pharmacy department [27]. Five studies used a 4% CHG liquid solution with rinsing [24, 42–45]. Two studies used a 2% CHG solution that was locally made. In one study, the 2% CHG solution was prepared by diluting Bactoshield chlorhexidine 4% Surgical Scrub (STERIS, Mentor, OH) [46]. The other study prepared 2% CHG solution by diluting bulk 4% CHG (Betasept; Purdue Pharma) 1:2 with tap water [47]. Another study used a 0.9% CHG solution in sterile water [48]. Nine studies used bundled interventions [24, 25, 28, 33, 35, 37, 43, 45, 48] incorporating other infection control interventions, such as reinforcement of hand hygiene during the study period, intranasal decolonization with mupirocin, and universal gloving and gowning by healthcare providers. The extent and timing of implementation of these interventions relative to CHG bathing were not described in detail in any of the studies. Details of characteristics of included studies are presented in Additional file 1: Table S1.

**Fig. 2:**
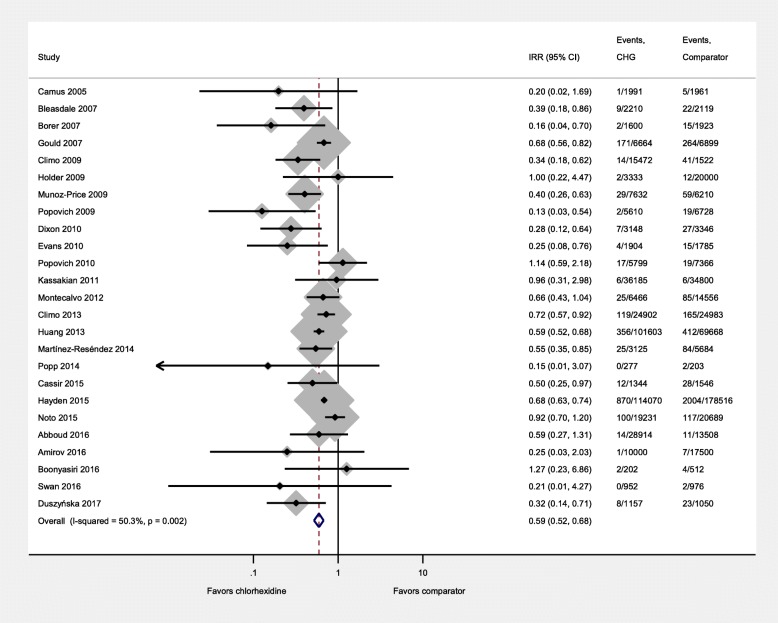
Forest plot showing that chlorhexidine bathing reduced the incidence of hospital acquired bloodstream infections; the dotted line indicates the mean estimated relative risk

There were 5259 HABSIs and 861,546 patient-days. Overall, the incidence rate of HABSI per 1000 patient days was 4.4 (95% confidence interval [CI]: 4.2–4.6) in the CHG group and 7.5 (95% CI: 7.3–7.8) in the comparator group. Figure 2 summarizes the effect of CHG bathing on HABSI. Sixteen studies made a distinction between CLABSI and other bloodstream infections. Among these studies, 75% of HABSI in the CHG group and 71% in the comparator group were CLABSIs. There was moderate heterogeneity in the effect of CHG bathing across studies (τ^2^ = 0.17; I^2^ = 50.3%, *p* = 0.002) [23]. The random-effects IRR for CHG bathing was 0.59 (95% CI: 0.52–0.68); in other words, the incidence rate of HABSIs was reduced by approximately 40% (95% CI: 32–48%).

The effect of CHG bathing was consistent within subgroups: randomized (0.67, 95% CI: 0.53–0.85) vs. non-randomized studies (0.54, 95% CI: 0.44–0.65), bundled (0.66, 95% CI: 0.62–0.70) vs. non-bundled interventions (0.51, 95% CI: 0.39–0.68), CHG impregnated wipes (0.63, 95% CI: 0.55–0.73) vs. CHG solution (0.41, 95% CI: 0.26–0.64), and ICU (0.58, 95% CI: 0.49–0.68) vs. non-ICU settings (0.56, 95% CI: 0.38–0.83) (Additional file 2: Figures S1-S4). A meta-regression analysis to examine differences between these subgroups showed that the stratified estimates did not significantly differ between subgroups (Additional file 3: Table S3).

Analysis using number of patients as the denominator for a subset of studies that provided this data did not change our findings (IRR = 0.59, 95% CI: 0.51–0.68, I^2^ = 60.5%, *p* < 0.001).

Only three studies reported all five measures of fidelity [34, 35, 37]. Twelve percent (3/26) of the studies reported four measures of fidelity, 15% (4/26) reported three measures, 27% (7/26) reported two measures, and 35% (9/26) reported one fidelity measure. The most frequently missed fidelity measures were participant responsiveness (21/26) and exposure or dose (17/26), while program differentiation was not missed by any study. Most studies (16/26, 62%) did not report how fidelity was assessed, and those that did reported measures used to assess adherence, quality of the delivery, and participant responsiveness (Additional file 4: Table S2).

Ten studies did not report any patient-centered outcomes. The remaining 16 studies described monitoring of adverse events to CHG as reported by patients and/or from medical records. Of these 16, eight studies reported data on adverse events, such as skin rashes, skin dryness, and pruritus [28, 31, 32, 35, 38, 43, 46, 47].

**Fig. 3:**
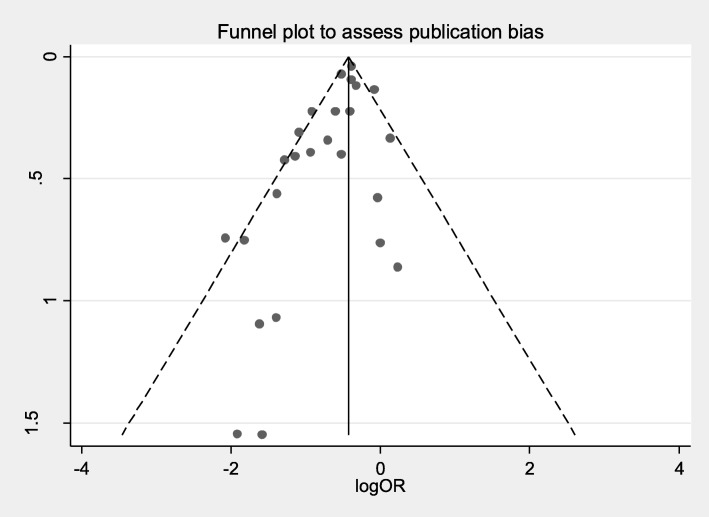
Funnel plot to assess publication bias

The risk of bias for each of the studies is reported in Additional file 1: Table S1. Overall, most of the studies were appraised as being at low risk of bias in the majority of the five domains. The main sources of potential bias came from allocation (pre-post designs), presence of potentially confounding interventions such as mupirocin nasal decolonization and non-standard definitions for infection. Two studies did not provide their definition for bloodstream infection [34, 47]. Although visual inspection of the funnel plot (Fig. 3) suggested the presence of publication bias, the Egger’s test did not show evidence of publication bias (Egger’s test, *p* = 0.80).

### Microbiology—HABSI causative organisms

Twenty-three studies (85%) reported data on the causative organisms of HABSIs. Beneficial impact of CHG on gram-positive bacteria other than CoNS was reported in ten (43%) studies; impact of CHG on CoNS in eight (35%) studies; impact of CHG on gram-negative bacteria in eight (35%) studies; and beneficial impact of CHG on fungi (*Candida* species) in five (22%) of the studies.

## Discussion

We found that CHG bathing of patients is associated with a consistent, clinically important, and statistically significant reduction in the risk of HABSIs. This effect was consistent across study settings, study designs, whether the intervention was bundled or not, and whether a no-rinse approach (i.e., CHG-impregnated wipes) or CHG solution requiring rinsing was used. The finding that the effect of CHG was present even with the rinsing approach was interesting because the literature suggests that rinsing results in lower levels of CHG on the skin [49, 50]. However, a certain amount of CHG remains even with rinsing, and this might account for the observed effect with rinsing [51].

There is considerable variation in the implementation of CHG bathing. Most CHG intervention studies included in our analysis failed to report measures of fidelity. Given the complexity of effectively implementing a behavioral intervention in healthcare settings, a systematic, careful assessment of fidelity is essential. The three studies that reported all fidelity components heavily engaged their frontline staff in rolling out the intervention and conducted direct observations of the bathing process [34, 35, 37]. Involvement of frontline staff in CHG bathing rollout has been shown to be important in ensuring success and sustainability of CHG bathing [14]. Many studies tended to report adherence and quality of the delivery fidelity measures. Although these are necessary for ensuring that the intervention is performed at a given facility, they are not sufficient for replication of the studies in other settings. Therefore, reporting adherence and quality of the delivery alone without the other three fidelity measures (i.e., exposure or dose, participant responsiveness and program differentiation) limits the generalizability of study findings.

As an increasing number of institutions adopt CHG bathing as an important horizontal pathogen-independent infection prevention strategy, standardization is an essential step. As most studies were not RCTs, thus precluding a robust assessment of causality, future research should focus on understanding and reporting factors that facilitate or impede high-fidelity implementation.

Our study has implications for clinical practice. Our results show that CHG bathing should be considered for adoption by institutions as part of a comprehensive HABSI reduction strategy that includes careful monitoring of adherence to the bathing protocol.

Our study extends the findings of previous reviews. Unlike other previous reviews [52–57], including two conducted by our group [6, 7], this analysis includes a rigorous assessment of implementation as well as patient-centered factors. We found that many studies did not report assessment of any patient outcomes, and those that did only reported adverse events, such as skin rashes, skin dryness, and pruritus. CHG bathing interventions should incorporate comprehensive assessment of patient-centered outcomes, such as patient comfort during the bath and perceptions regarding the smell and feel of the chosen product in addition to adverse cutaneous effects – all of these factors have been reported to affect patient acceptance of CHG bathing [14]. This is especially important in light of the recent FDA advisory on the risk of rare allergic skin reactions to CHG [58].

Our study also showed that CHG bathing impacted all microorganisms responsible for HABSIs as expected from the broad-spectrum nature of CHG [59]. One of the very few negative ICU studies that did not show an effect of CHG bathing had serious limitations [39]. The study was a single-center unblinded study that used a composite endpoint of all HAIs rather than HABSIs for which there is a high biological plausibility for CHG’s role in preventing infections.

Although not assessed in our study, CHG cost is an important factor that warrants mention. CHG bathing can lead to substantial cost savings for institutions in which it is implemented. A cost analysis by Holder & Zellinger et al. showed that implementing CHG bathing in all ICUs of 93-ICU-bed hospital would save the hospital $1.56 million per year [34]. Dixon and Carver showed implementing CHG bathing in a nine-bed surgical ICU compared to ordinary soap and water was associated with $728,820 in cost savings over a 17-month intervention period. [30]. These studies suggest that considering the potentially prevented HAIs, the use of CHG is associated with cost savings.

Another important consideration before implementing CHG is the potential of development of bacterial resistance to CHG. Examination of current literature indicates that the evidence for the development of resistance to CHG has been mixed. An eight-year prospective study of MRSA in a surgical ICU showed a trend towards increasing prevalence of the resistance gene *qacA/B* [60]. Another ICU study showed that 2 and 7% of MRSA isolates were *qacA/B* and *smr* positive, respectively [61]. Using published data from clinical isolates and comparing their CHG minimum inhibitory concentrations with epidemiological cut-off values, Kampf showed CHG resistance by certain bacteria but not others [11]. On the other hand, a multicenter cluster randomized ICU study did not show CHG resistance [29]. Therefore, there is still clinical equipoise regarding bacterial resistance to CHG.

Our study has limitations. As this meta-analysis focused on adult patients by design, its findings may not be applicable to pediatric populations. There was a high degree of heterogeneity in the studies included in our analysis. We observed that heterogeneity came mainly from studies that were non-bundled rather than from bundled ones. In contrast to our findings, previous studies have shown that bundled interventions have a greater effect on the incidence of CLABSIs than non-bundled interventions [62, 63]. The heterogeneity observed with regard to non-bundled interventions and the fact that our study pooled all HABSIs could explain this difference. Infection control “care bundles,” such as the Institute for Healthcare Improvement Central Line Bundle [64], have clearly defined bundle components and are more likely to be implemented in a similar fashion across facilities. A high degree of compliance is needed for bundled interventions to be effective [65]. For CHG studies (both bundled and non-bundled), there is likely to be variation in CHG bathing processes, products used, and populations under study. We employed a random-effects model and explored potential causes of heterogeneity in depth. In some studies, CHG bathing was instituted as a quality improvement intervention in combination with other concurrent interventions, such as screening for resistant bacteria [24, 32, 42] or reinforcement of hand hygiene practices [37, 43–45]. The independent impact of these concurrent interventions cannot be accounted for in our meta-analysis. Another limitation is that the included studies defined CLABSIs differently and most did not provide information on the site of origin. Only a few studies reported bloodstream as a source of the HABSI, in which case data for bloodstream was extracted. In addition, we could not rank interventions based on their fidelity measurements, because no published studies examined which of the five elements are most important. Some are more likely to influence study replicability (dose, differentiation, quality), while others are more likely to influence intervention sustainability (participant responsiveness, adherence) [20, 66]. Regardless, interventions should aim at incorporating all five, and future research should examine the importance of each element individually.

## Conclusion

We found that patient bathing with CHG significantly reduced the incidence of HABSIs in both ICU and non-ICU settings. However, the strength of evidence for non-ICU use was lower. As a horizontal infection prevention strategy that covers a broad spectrum of pathogens, CHG bathing is an effective, relatively low-cost intervention that should be implemented with high fidelity to achieve maximum impact. For sustainability and replicability essential for effective implementation, fidelity assessment that goes beyond whether a patient received an intervention or not should be standard practice particularly for complex behavioral interventions such as CHG bathing.

## Additional files

Additional file 1: **Table S1.** Characteristics of included studies. (DOCX 57 kb)

Additional file 2: **Figure S1.** Effect of chlorhexidine gluconate bathing comparing Randomized vs. non-randomized studies. **Figure S2.** Effect of chlorhexidine gluconate bathing comparing bundled vs. non-bundled interventions. **Figure S3.** Effect of chlorhexidine gluconate bathing comparing 2% chlorhexidine impregnated wipes vs. 4% CHG solution. **Figure S4.** Effect of chlorhexidine gluconate bathing comparing intensive care unit (ICU) vs. non-ICU settings. (PDF 164 kb)

Additional file 3: **Table S3.** Meta-regression analysis showed that the stratified estimates did not significantly differ between subgroups. (DOCX 14 kb)

Additional file 4: **Table S2.** Fidelity assessment. (DOCX 47 kb)

## Abbreviations

CHG: Chlorhexidine gluconate
CI: Confidence interval
CLABSI: Central line-associated bloodstream infections
CoNS: Coagulase-negative staphylococci
CRT: Cluster randomized trials
FDA: Food and Drug Administration
HABSIs: Hospital-acquired bloodstream infections
HAIs: Healthcare-associated infections
ICU: Intensive care unit
IRR: Incidence rate ratio
RCT: Randomized controlled trials
