# Supplementary material for: The impact of chlorhexidine bathing on hospital-acquired bloodstream infections: a systematic review and meta-analysis
Source: BMC Infect Dis. 2019 May 14;19:416. doi: 10.1186/s12879-019-4002-7 (PMC6518712; doi:10.1186/s12879-019-4002-7)
Supplement: Supplementary file 1 — Table S1. Characteristics of included studies. (DOCX 57 kb) [file 12879_2019_4002_MOESM1_ESM.docx]

**Additional file 1: Table S1** Characteristics of included studies

| **Study** | **Study design** | **Country** | **Setting** | **Study duration** | **Number of subjects (N)** | **Mean age, years (SD)** | **Subject Gender (% male)** | **Intervention** | **Comparator** | **Bundled intervention** | **Risk of bias** |
| --- | --- | --- | --- | --- | --- | --- | --- | --- | --- | --- | --- |
| Camus 2005 [43] | Randomized clinical trial | France | Multisite, three medical ICUs | Apr 1996 - Oct 1998 | CHG: 130  Control: 126 | CHG^a^: 65 (21-86)  Control: 67 (19-84) | CHG: 56%  Control: 56% | Twice a day 4% CHG solution baths (Hibiscrub; Astra-Zeneca, Rueil- Malmaison, France) | Twice a day bathing with plain soap and water | Yes | Low risk: 5 domains  High risk: None |
| Bleasdale 2007 [8] | Prospective, 2-arm, crossover (i.e., concurrent control group) clinical trial | USA | Single center, two medical ICUs | June 2005 – June 2006 | CHG: 391  Control: 445 | CHG: 53 (16)  Control: 52 (15) | CHG: 60%  Control: 60% | Daily bathing with 2% CHG-impregnated washcloths (Sage 2% CHG cloths; Sage Products Inc, Cary, Illinois) | Daily bathing with plain soap and water | No | Low risk: 4 domains  High risk: 1 domain |
| Borer 2007 [42] | Before and after quasi-experimental study | Israel | Single center medical ICU | March 2002 - December 2003 | CHG: 320  Control: 329 | NR | NR | Daily bathing with 4% CHG (Septal Scrub, Teva Medical, Ashdod Israel) | Daily bathing with plain soap and water | No | Low risk: 1 domain  High risk: 4 domains |
| Gould 2007 [45] | Before and after quasi-experimental study | USA | Single center, 16-bed mixed medical-surgical ICU | May 1999–Apr 2001 and May 2001–Apr 2003 | CHG: 1421  Control: 1232 | CHG: 58.5  Control: 56.8 | CHG: 46%  Control: 56% | Daily bathing with 4% CHG solution (SSL International, Manchester, UK) | Daily bathing with plain soap and water | Yes | Low risk: None domains  High risk:5 domains |
| Climo 2009 [44] | Before– after quasi-experimental design | USA | Multicenter,6 ICUs (coronary care units, surgical, cardiac surgery, mixed) | December 2004 - January 2006 | CHG: 2650  Control: 2670 | NR | NR | Daily 4% CHG solution | Daily bathing with plain soap and water | No | Low risk: 3 domains  High risk: 2 domains |
| Holder 2009 [34] | Before and after quasi-experimental study | USA | Multicenter, MICUs of 5 hospitals | Oct 2007–Mar 2008 and Apr 2008–Sep 2008 | CHG: NR (20,000 patient-days)  Control: NR (3,333 patient days) | NR | NR | Daily bathing with 2% CHG impregnated cloths | Daily bathing with plain soap and water | No | Low risk: None domains  High risk:5 domain |
| Munoz-Price 2009 [47] | Quasi-experimental design (3-phase design) | USA | Single center, 70-bed LTACH | February 2006 to February 2008 | CHG: 405  Control: 340 | NR | NR | Daily bathing with 2% CHG solution prepared by pharmacy department by diluting bulk 4% chlorhexidine (Betasept; Purdue Pharma) | Weekly bathing with or plain wash cloths (Comfort Baths; Sage Products) | No | Low risk: 3 domains  High risk: 2 domains |
| Popovich 2009 [40] | Before and after quasi-experimental study | USA | Single center, 21-bed MICU | Sep 2004–Oct 2006 | CHG: 1951  Control: 2163 | CHG: 59.3  Control: 59.5 | NR | Daily bathing with no rinse 2% CHG (Sage Products, Cary, IL) | Daily bathing with plain soap and water | No | Low risk: 4 domains  High risk: 1 domains |
| Dixon 2010 [30] | Observational cohort study using historical controls | USA | Single center,9-bed SICU | Jan 2007–Sep 2009 | CHG: NR (272,779 patient-days)  Control: NR (146547 patient days) | NR | NR | Daily bathing with no rinse 2% CHG (Sage Products, Cary, IL) | Daily bathing with plain soap and water | No | Low risk: 3 domains  High risk: 2 domains |
| Evans 2010 [32] | Before and after quasi-experimental study (analyzed retrospectively) | USA | Single center, 12-bed trauma ICU | Nov 2006–Apr 2007 and May–Oct 2007 | CHG: 286  Control: 253 | CHG: 39 (16)  Control: 40 (15) | CHG: 73.8%  Control: 68.8% | Daily bathing with no rinse 2% CHG (Sage Products, Cary, IL) | Daily bathing with washcloths without chlorhexidine(Impreva Bath; Sage Products Inc, Cary, Illinois) | No | Low risk: 4 domains  High risk:1 domain |
| Popovich 2010 [41] | Before and after quasi-experimental study | USA | Single center, 30-bed surgical IC | Sep 2004–Oct 2006 | CHG: 1,938  Control: 1,387 | CHG: 59.2  Control: 58.4 | NR | Daily bathing with no rinse 2% CHG (Sage Products, Cary, IL) | Daily bathing with plain soap and water | No | Low risk: 4 domains  High risk: 1 domains |
| Kassakian 2011 [36] | Before and after quasi-experimental study | USA | Single center, 4 adult general medicine units | January to December 2008 and February 2009 to March 2010 | CHG: 7,699  Control: 7,102 | CHG: 60.7  Control: 61.5 | CHG: 47%  Control: 47% | Daily bathing with no rinse 2% CHG cloths (Sage Products, Cary, IL) | Daily bathing with plain soap and water | No | Low risk: 3 domains  High risk: 2 domains |
| Montecalvo 2012 [38] | Quasi-experimental design (3-phase design) | USA | Multicenter, MICU and respiratory care unit of a tertiary care hospital and the medical-surgical ICU of 4 community hospitals | April 2008 to August 2010 | CHG: NR (6,466 device days)  Control: NR (14,566 device days) | NR | NR | Daily bathing with no rinse 2% CHG (Sage Products, Cary, IL) and twice-daily intranasal mupirocin for 5 days | Daily bathing with plain soap and water or plain wash cloths | No | Low risk: 4 domains  High risk: 1 domains |
| Climo 2013 [29] | Cluster-randomized, non-blinded crossover trial | USA | Multicenter, 9 ICUs (4 SICUs, 3 MICUs, 1 CSICU, 1 MICU-CCU, 1 BMT unit) | August 2007 - February 2009 | CHG: 3,970  Control: 3,842 | NR | NR | Daily bathing with no rinse 2% CHG cloths (Sage Products, Cary, IL) | Daily bathing with non-antimicrobial washcloths (Comfort Bath, Sage Products) | No | Low risk: 5 domains  High risk: None |
| Huang 2013 [35] | Pragmatic cluster-randomized trial | USA | Multicenter, 72 adult ICUs from 43 hospitals | January 2009 to September 2011 | CHG: 26024  Control: 17,356 | NR | CHG: 53%  Control:53% | Daily bathing with no rinse 2% CHG (Sage Products, Cary, IL) and twice-daily intranasal mupirocin for 5 days | MRSA screening and isolation and decolonization of MRSA carriers with twice-daily intranasal mupirocin for 5 day | Yes | Low risk: 5 domains  High risk: None |
| Martínez-Reséndez 2014 [37] | Quasi-experimental design (3-phase design) | Mexico | Single center, 2 adult 10-bed medical-surgical ICUs | January 2012 to June 2013 | CHG: 327  Control: 680 | CHG: 49.7  Control: 47.8 | CHG: 60.2%  Control: 66.3% | Daily bathing with 2% CHG-impregnated wipes (Clorhexi-Wipes One Step; G70 Antisepsis, Leon, Mexico) | Daily bathing with plain soap and water | Yes | Low risk: 4 domains  High risk: 1 domains |
| Popp 2014 [48] | Quality improvement project with before and after data | USA | Single center 8-bed burns unit | January 2010 - June 2012 | CHG: 277  Control: 203 | NR | NR | Twice daily bathing with 0.9% CHG solution in sterile water | Twice daily bathing with plain soap and water | No | Low risk: 3 domains  High risk: 2 domains |
| Cassir 2015 [28] | Quasi-experimental design with a control and intervention period | France | Single center, 14-bed medical ICU | March 2012 - May 2013 | CHG: 150  Control: 175 | CHG^a^: 58 (46-68)  Control: 61 (48-73) | CHG: 61.3%  Control: 58.2% | Daily bathing with no rinse 2% CHG cloths (Sage Products, Cary, IL) | Daily bathing with plain soap and water | Yes | Low risk: 3 domains  High risk: 2 domains |
| Hayden 2015 [33] | Stepped-wedge design with pre-intervention and intervention period | USA | Multicenter, medical and high-acuity units of 7 LTACHs | February 2010 and June 2013 | CHG: 3738  Control: 5282 | CHG: 64 (16)  Control: (63 (16) | CHG: 55%  Control:55% | Daily bathing with no rinse 2% CHG cloths (Sage Products, Cary, IL) | Daily bathing with plain soap and water | Yes | Low risk: 3 domains  High risk: 2 domains |
| Noto 2015 [39] | Cluster-randomized, cross-over study | USA | Single center, five adult ICUs (34-bed neurological, 34-bed surgical, 31 bed-trauma, 27-bed cardiovascular and 34-bed medical) | July 2012 - July 2013 | CHG: 4488  Control: 4852 | CHG^a^: 56.0 (42-68)  Control:57.0 (42–68) | CHG: 57.6%  Control: 57.8% | Daily bathing with no rinse 2% CHG (Sage Products, Cary, IL) | Daily bathing with or plain wash cloths (Comfort Bath, Sage Products, Cary, IL) | No | Low risk: 4 domains  High risk: 1 domains |
| Willis 2015 [24] | Quality improvement project using a quasi-experimental design | Singapore | Single center, general wards | 2011 - 2013 | CHG: 4598  Control: 65401 | NR | NR | Daily bathing with 4% CHG soap | Daily bathing with plain soap and water | Yes | Low risk: 1 domains  High risk: 5 domains |
| Abboud 2016 [25] | Before and after quasi-experimental study | Brazil | Single center, cardiac ICU | April 2013 - December 2014 | CHG: 1799  Control: 764 | NR | NR | Daily bathing with no rinse 2% CHG cloths (Sage Products, Cary, IL) | Daily bathing with plain soap and water | Yes | Low risk: 3 domains  High risk: 2 domains |
| Amirov 2016 [26] | Cluster-randomized trial | Canada | Single center, 3 geriatric chronic care units | 12-months (year not specified) | CHG: 35  Control: 87 | CHG^a^: 88  Control: 89 | CHG: 51.5%  Control: 48.5% | Daily bathing with no rinse 2% CHG cloths (Sage Products, Cary, IL) | Daily bating with non-antiseptic washcloths (Comfort Bath; Sage Products) | No | Low risk: 5 domains  High risk: None |
| Boonyasiri 2016 [27] | Randomized, open-label controlled trial | Thailand | Single center, four medical ICUs | December 2013 - January 2015 | CHG: 189  Control: 199 | CHG: 65 (17.9)  Control: 67 (17.2) | CHG: 44.4%  Control: 46.2% | 2% chlorhexidine-impregnated washcloths, locally made | Plain soap | No | Low risk: 4 domains  High risk:1 domain |
| Swan 2016 [46] | Pragmatic randomized trial | USA | Single center, 24–bed surgical ICU | July 2012-May 2013 | CHG: 161  Control: 164 | CHG: 59.4 (15.9)  Control: 60.2 (16.5) | CHG: 61%  Control: 52% | Every other day bathing with 2% CHG solution created by mixing 8 oz  of warm tap water with 8 oz (two 4-oz bottles) of Bactoshield  Chlorhexidine 4% Surgical Scrub (STERIS, Mentor, OH). | Daily bathing with soap and water | No | Low risk: 5 domains  High risk: None |
| Duszyńska 2017 [31] | Observational, prospective study | Poland | Mixed 16-bed tertiary ICU | Jan 2014-June 2015 | CHG: 105  Control: 92 | CHG: 62 (18)  Control: 64 (18) | CHG:60%  Control: 60% | Daily bathing with no rinse 2% CHG (Sage Products, Cary, IL) | Daily bathing with plain soap and water | No | Low risk: 4 domains  High risk:1 domain |

a Age reported as median. NR= Not reported. LTACH=Long-term acute care hospital. SICU=Surgical intensive care unit. MICU= Medical intensive care unit. BMT= Bone marrow transplant. CCU=coronary care unit. CSICU=cardiac surgery intensive care unit. CHG=Chlorhexidine gluconate.
