# Supplementary material for: The impact of chlorhexidine bathing on hospital-acquired bloodstream infections: a systematic review and meta-analysis
Source: BMC Infect Dis. 2019 May 14;19:416. doi: 10.1186/s12879-019-4002-7 (PMC6518712; doi:10.1186/s12879-019-4002-7)

FIGURES FOR SUBGROUP ANALYSES

Figure 1. Effect of chlorhexidine gluconate bathing comparing Randomized vs. non-randomized studies

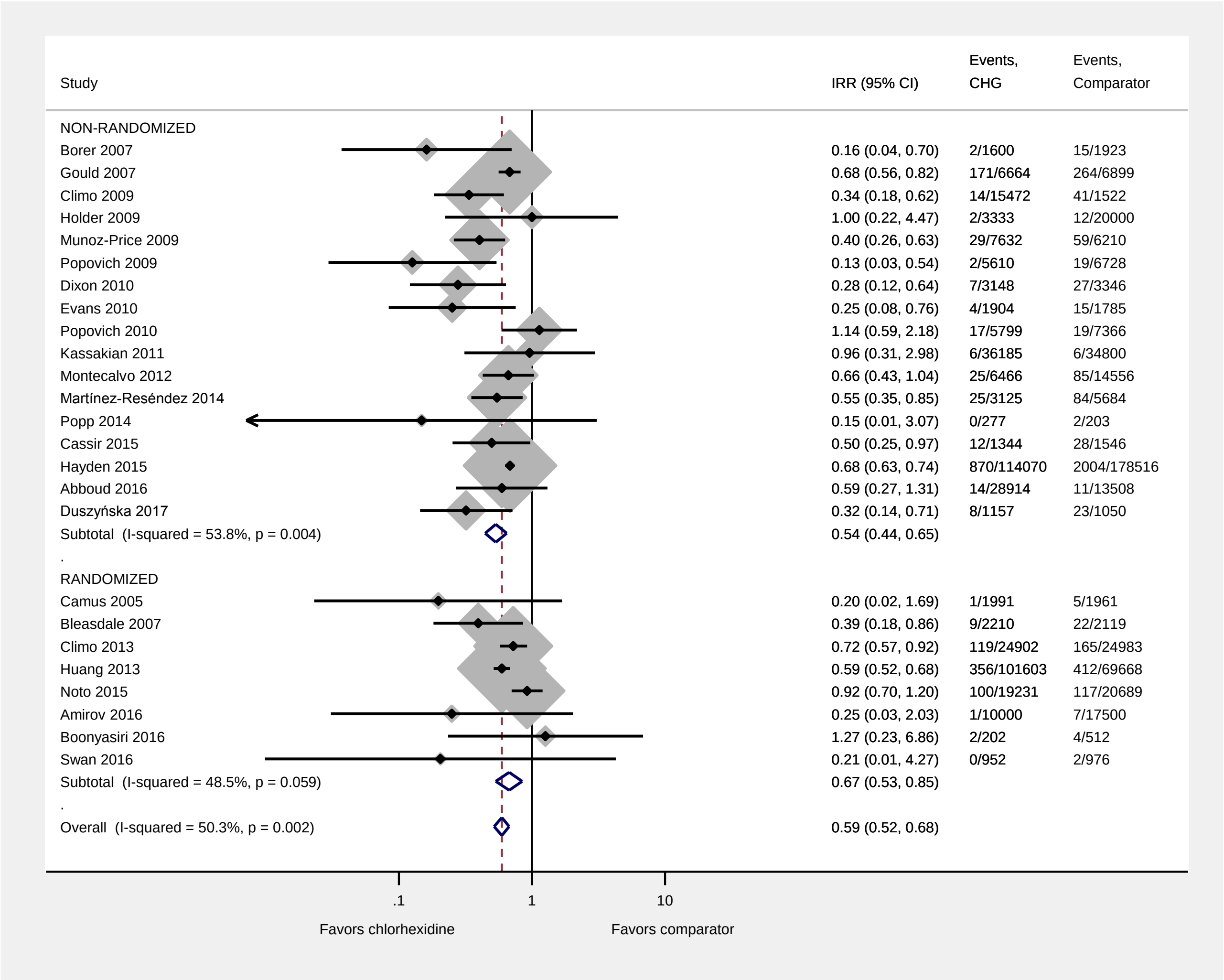

Figure 2. Effect of chlorhexidine gluconate bathing comparing bundled vs. non-bundled interventions

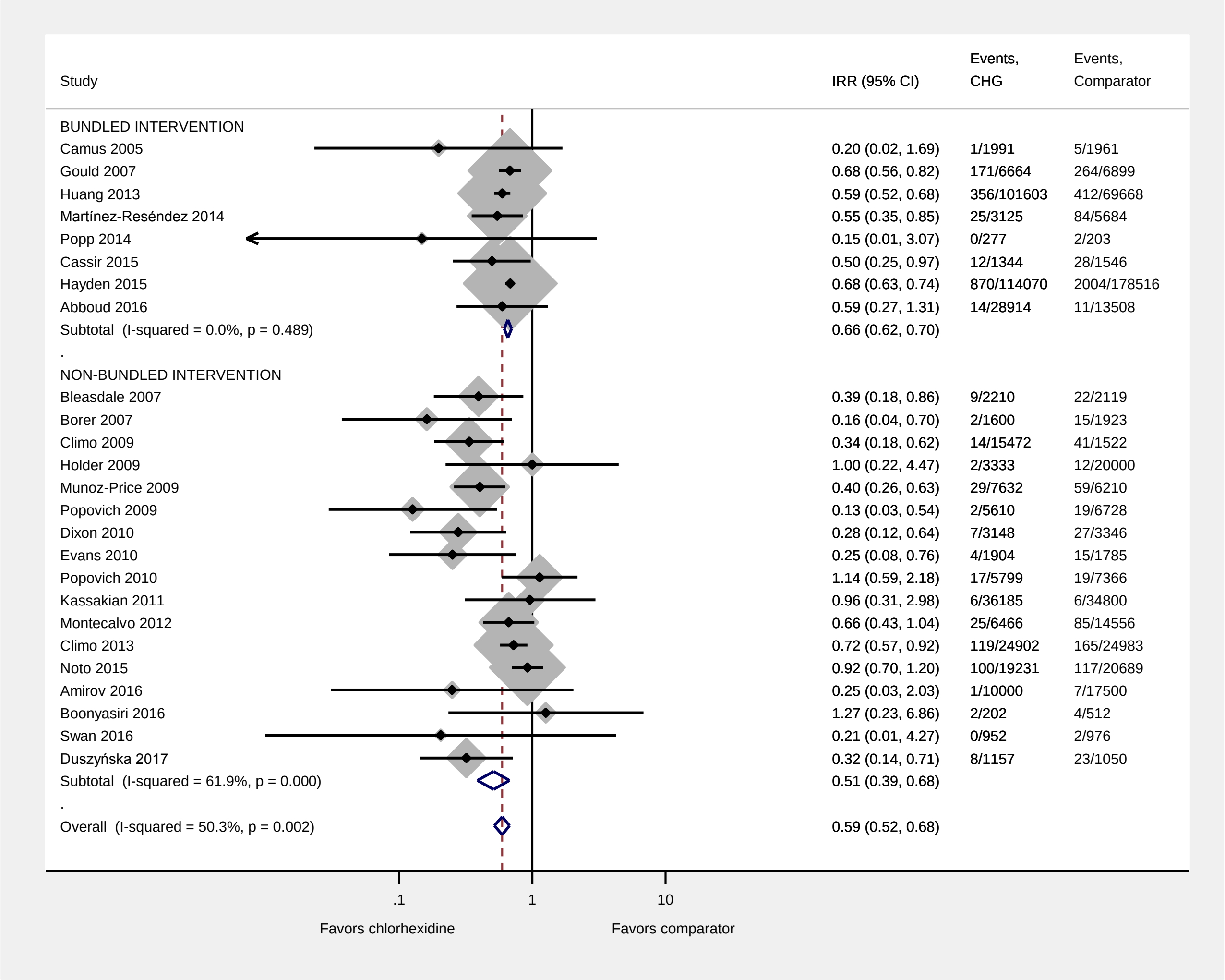

Figure 3. Effect of chlorhexidine gluconate bathing comparing 2% chlorhexidine impregnated wipes vs. 4% CHG solution

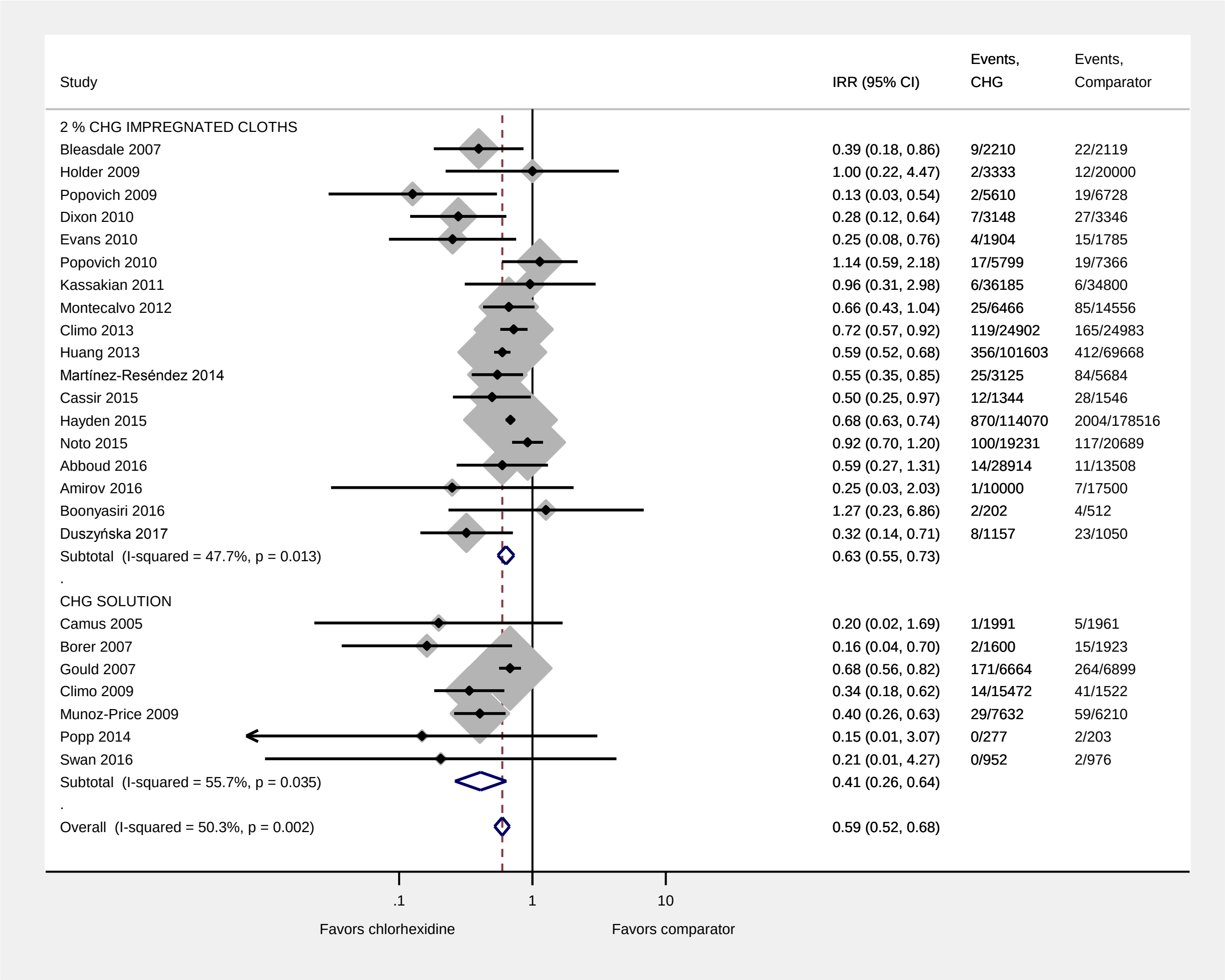

Figure 4. Effect of chlorhexidine gluconate bathing comparing intensive care unit (ICU) vs. non-ICU settings

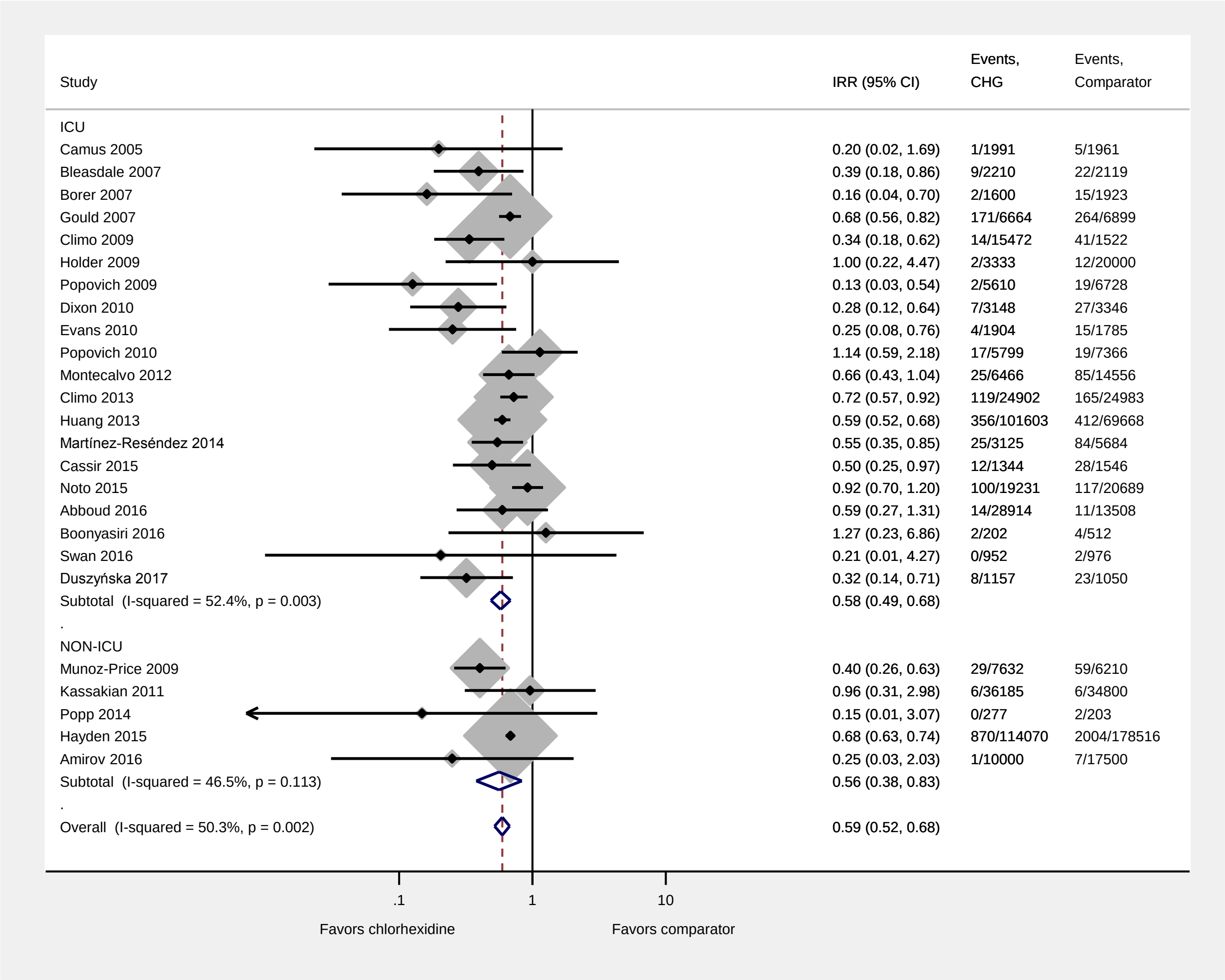

Supplement: Supplementary file 2 — Figure S1. Effect of chlorhexidine gluconate bathing comparing Randomized vs. non-randomized studies. Figure S2. Effect of chlorhexidine gluconate bathing comparing bundled vs. non-bundled interventions. Figure S3. Effect of chlorhexidine gluconate bathing comparing 2% chlorhexidine impregnated wipes vs. 4% CHG solution. Figure S4. Effect of chlorhexidine gluconate bathing comparing intensive care unit (ICU) vs. non-ICU settings. (PDF 164 kb) [file 12879_2019_4002_MOESM2_ESM.pdf]
