# Supplementary material for: The impact of chlorhexidine bathing on hospital-acquired bloodstream infections: a systematic review and meta-analysis
Source: BMC Infect Dis. 2019 May 14;19:416. doi: 10.1186/s12879-019-4002-7 (PMC6518712; doi:10.1186/s12879-019-4002-7)
Supplement: Supplementary file 3 — Table S3. Meta-regression analysis showed that the stratified estimates did not significantly differ between subgroups. (DOCX 14 kb) [file 12879_2019_4002_MOESM3_ESM.docx]

**Additional file 3: Table S3**. Meta-regression analysis showed that the stratified estimates did not significantly differ between subgroups.

| Characteristic | exp(β) | Standard Error | t | P>\|t\| | [95% Confidence Interval] |
| --- | --- | --- | --- | --- | --- |
| randomized vs. non-randomized studies | 1.25 | .231 | 1.19 | 0.245 | .850 - 1.83 |
| bundled vs. non-bundled studies | 1.11 | .208 | 0.58 | 0.568 | .757 - 1.64 |
| 2% chlorhexidine impregnated wipes vs. 4% CHG solution | 1.37 | .287 | 1.49 | 0.150 | .886 - 2.11 |
| ICU vs. no-ICU settings | 1.00 | .239 | 0.00 | 0.998 | .610 - 1.64 |
