# Supplementary material for: The impact of chlorhexidine bathing on hospital-acquired bloodstream infections: a systematic review and meta-analysis
Source: BMC Infect Dis. 2019 May 14;19:416. doi: 10.1186/s12879-019-4002-7 (PMC6518712; doi:10.1186/s12879-019-4002-7)
Supplement: Supplementary file 4 — Table S2. Fidelity assessment. (DOCX 47 kb) [file 12879_2019_4002_MOESM4_ESM.docx]

**Additional file 4: Table S2.** Fidelity assessment

| **Study** | **Source of fidelity measures (observations/self-report)** | **Instrument for data collection (checklist/ survey forms and logs)** | **Fidelity measure(s) assessed (adherence, exposure/dose, quality, responsiveness, differentiation)** ^a^ | **Number of Fidelity measure(s) missed** |
| --- | --- | --- | --- | --- |
| Camus 2005 [43] | Not reported | Not reported | Differentiation | 4 |
| Bleasdale 2007 [8] | Not reported | Not reported | Differentiation | 4 |
| Borer 2007 [42] | Not reported | Not reported | Differentiation | 4 |
| Gould 2007 [45] | Not reported | Not reported | Differentiation | 4 |
| Climo 2009 [44] | Process measures | Inventory of chlorhexidine bottles supplied to the study units | Adherence, differentiation | 3 |
| Holder 2009 [34] | Process measures | Documentation of bathing in patient records | Adherence, exposure/dose, quality, responsiveness, differentiation | 0 |
| Munoz-Price 2009 [47] | Not reported | Not reported | Differentiation | 4 |
| Popovich 2009 [40] | Not reported | Not reported | Differentiation, exposure/dose | 3 |
| Dixon 2010 [30] | Observations (nursing staff) | Checklist | Adherence, differentiation, quality | 2 |
| Evans 2010 [32] | Not reported | Not reported | Differentiation | 4 |
| Popovich 2010 [41] | Not reported | Not reported | Differentiation, exposure/dose | 3 |
| Kassakian 2011 [36] | Process measures | Unit census numbers and hospital purchasing records | Adherence, differentiation | 3 |
| Montecalvo 2012 [38] | Process measures | Bathing log | Adherence, exposure, differentiation, quality | 1 |
| Climo 2013 [29] | Not reported | Not applicable | Differentiation, exposure | 3 |
| Huang 2013 [35] | Observations (Nursing directors) and process measures | Bathing logs and documentation of bathing in patient records | Adherence, exposure/dose, quality, responsiveness, differentiation | 0 |
| Martínez-Reséndez 2014 [37] | Observations (Head nurse and unit nurse managers) | Bathing log | Adherence, exposure/dose, quality, responsiveness, differentiation | 0 |
| Popp 2014 [48] | Not reported | Not reported | Differentiation | 4 |
| Cassir 2015 [28] | Not reported | Not reported | Differentiation | 4 |
| Hayden 2015 [33] | Observations (infection preventionists) | Checklist | Adherence, quality, differentiation, exposure/dose | 1 |
| Noto 2015 [39] | Not reported | Not reported | Differentiation | 4 |
| Willis 2015 [24] | Not reported | Not reported | Adherence, differentiation | 3 |
| Abboud 2016 [25] | Not reported | Audits of the CHG bathing process | Adherence, quality, differentiation | 2 |
| Amirov 2016 [26] | Not reported | Bathing logs and audits of the CHG bathing process | Adherence, quality, differentiation | 2 |
| Boonyasiri 2016 [27] | Observations (nursing staff) | Not reported | Adherence, quality, differentiation | 2 |
| Swan 2016 [46] | Not reported | Not reported | Adherence, exposure/dose, quality, differentiation | 1 |
| Duszyńska 2017 [31] | Self-report by nursing staff | Questionnaire | Differentiation, responsiveness | 3 |

a Adherence= the extent to which the implemented program elements align with the intervention as outlined in the protocol (was the intervention performed?). Exposure or dose= how much of the program content reached the intended participants. Quality= the processes and content ideals of an intervention. Responsiveness= level of engagement of participants in an intervention including their view of the intervention. Differentiation= the uniqueness of the intervention.
